# Pacing-Induced Cardiomyopathy Following Permanent Pacemaker Implantation: A Case Report and Review of Management Strategies

**DOI:** 10.7759/cureus.90624

**Published:** 2025-08-20

**Authors:** Sameera Ihala Gamage, Tiron Chathuranga, Niroshana Dahanayake

**Affiliations:** 1 General Medicine, National Hospital Galle, Galle, LKA; 2 Department of Medicine, Faculty of Medicine, University of Ruhuna, Galle, LKA

**Keywords:** electrical and mechanical dyssynchrony, heart failure, pacing-induced cardiomyopathy, pleural effusion, right ventricular pacing

## Abstract

Pacing-induced cardiomyopathy (PICM) is a well-documented complication of long-term right ventricular pacing (RVP) leading to left ventricular systolic dysfunction and heart failure.

We describe a 58-year-old woman with a history of complete heart block who underwent single-chamber pacemaker implantation. Eleven months later, she presented with New York Heart Association (NYHA) class IV heart failure and bilateral pleural effusions. Investigations revealed a significant drop in ejection fraction from 55% to 25%, with no other identifiable cause of heart failure. She was diagnosed with PICM and successfully managed with medical therapy and upgrade to cardiac resynchronisation therapy pacemaker (CRT-P). Her clinical symptoms and left ventricular function improved significantly.

This case highlights the importance of early recognition of PICM and the benefit of CRT-P upgrade in patients with high RVP burden.

## Introduction

Pacing-induced cardiomyopathy (PICM) is a recognised complication arising from prolonged right ventricular pacing (RVP), especially in patients with a high ventricular pacing burden [1,2]. PICM is conventionally defined as a reduction in left ventricular ejection fraction (LVEF) attributable to chronic right ventricular pacing following permanent pacemaker implantation [3]. High ventricular pacing burden lacks a universally accepted definition, but has been variably described in the literature as ventricular pacing comprising ≥20%, ≥40%, or ≥60% of total beats, each threshold being associated with an increased risk of PICM in patients with preserved baseline ejection fraction [4].

The reported incidence of PICM ranges from 6% to 22% within three to 16 years post-implantation, with the variability largely influenced by heterogeneity in diagnostic thresholds, study populations, and follow-up durations across clinical investigations [5].

The dyssynchronous electrical activation caused by RVP results in mechanical inefficiency, ultimately contributing to the deterioration of left ventricular systolic function [6]. With the increasing prevalence of permanent pacemaker (PPM) implantation for atrioventricular block and other bradyarrhythmias, the incidence of PICM has become more clinically relevant. Despite advancements in pacing techniques and device programming, the early identification of patients at risk and the development of effective management strategies remain challenging in routine clinical practice.

## Case presentation

The patient was a 58-year-old woman with a past medical history significant only for the implantation of a single-chamber VVIR pacemaker for symptomatic complete heart block. Her pre-implantation echocardiogram, performed two weeks prior to device insertion, demonstrated normal cardiac chamber dimensions, normal valves, and a LVEF of 65%. Following implantation, she experienced good functional recovery, achieving New York Heart Association (NYHA) class I status and resuming normal daily activities.

**Figure 1:**
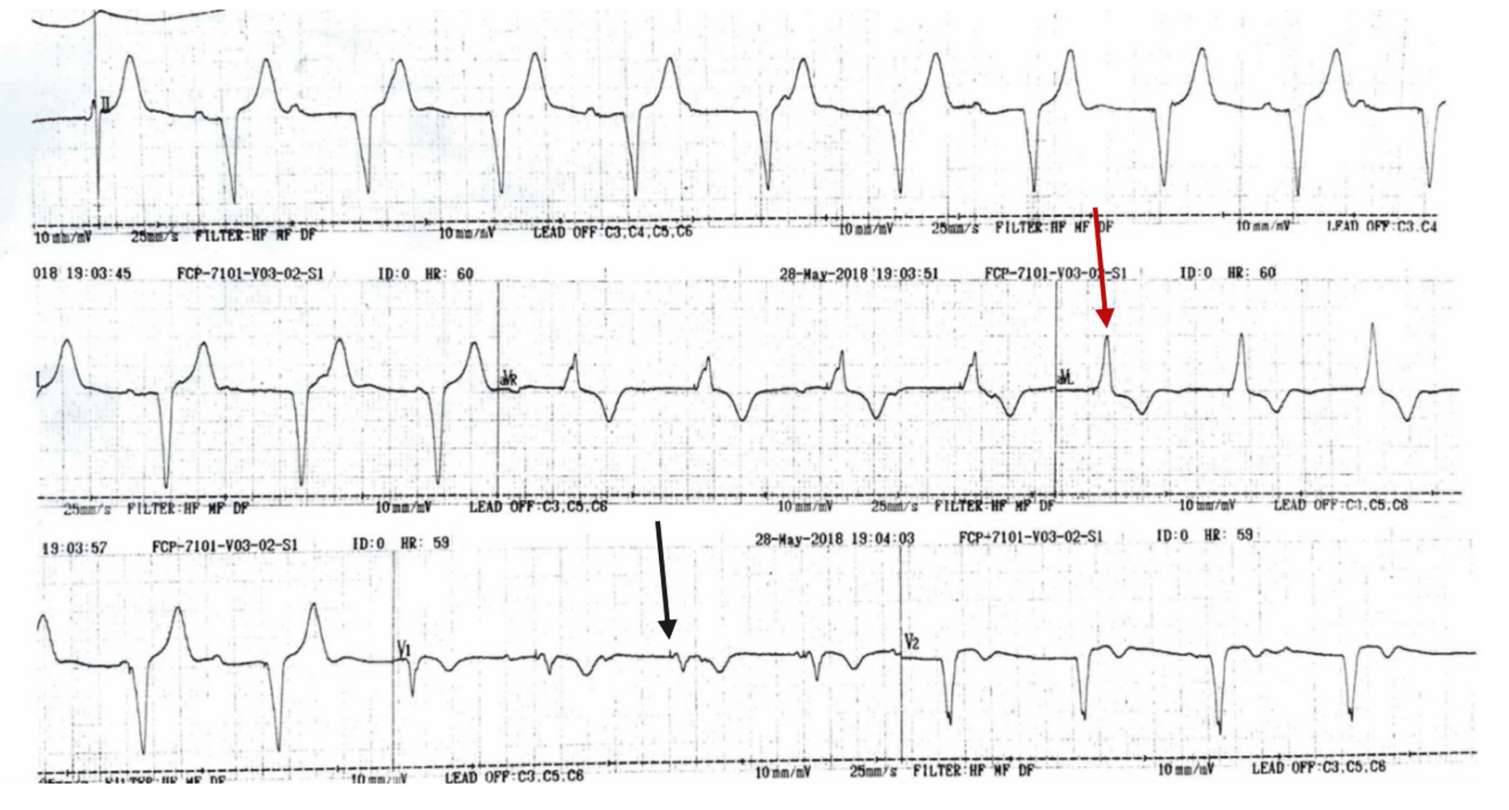
12-lead electrocardiogram demonstrating right ventricular paced rhythm The ECG shows a ventricular paced rhythm with visible pacemaker spikes preceding each QRS complex (black arrow). The QRS complexes are broad (>120 ms) with a left bundle branch block (LBBB) morphology, consistent with right ventricular pacing (red arrow).

**Figure 2:**
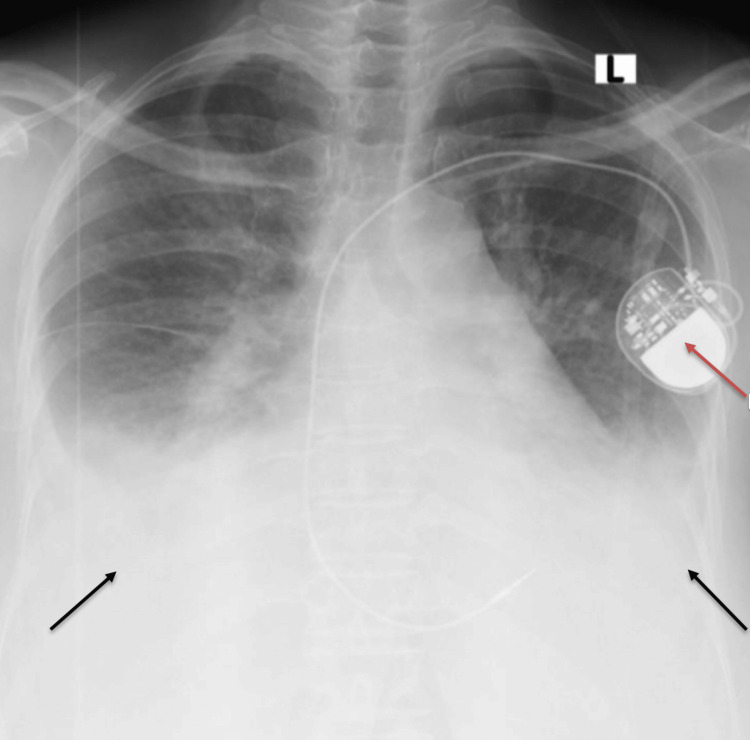
Frontal chest radiograph of the patient The image shows a single-chamber pace maker in situ (red arrow). Bilateral lower zone opacities (black arrows) are noted with obliteration of the costophrenic angles and diaphragmatic borders, consistent with bilateral pleural effusions.

Nearly one year later, she developed progressive dyspnoea and orthopnoea over six weeks prior to presentation, accompanied by pleuritic chest pain. She denied systemic features such as fever, weight loss, or constitutional symptoms. Examination revealed signs of biventricular heart failure. Device interrogation demonstrated a RVP burden of 96%. Electrocardiography showed a paced rhythm (Figure 1). N-terminal pro-B-type natriuretic peptide (NT-proBNP) was markedly elevated at 30,966 pg/mL. High-sensitivity troponin T was 22 ng/L (reference <14 ng/L), erythrocyte sedimentation rate (ESR) 8 mm/h, and C-reactive protein (CRP) 2 mg/L. Autoimmune and viral serologies were negative. Chest X-ray revealed bilateral pleural effusions (Figure 2). Pleural fluid analysis confirmed transudative characteristics (serum protein 40g/l, pleural protein 22 g/L, pleural lactate dehydrogenase (LDH) 120 U/L) with no malignant cells. Echocardiography showed an LVEF of 25-30%, representing a substantial decline from her pre-implantation value. 

Extensive evaluation was undertaken to exclude other causes of newly developed heart failure. Ischemic cardiomyopathy was ruled out, as coronary angiography prior to pacemaker implantation demonstrated unobstructed coronary arteries. Valvular heart disease was present but not hemodynamically significant enough to account for the severity of symptoms. Myocarditis and infiltrative cardiomyopathies were considered; however, cardiac magnetic resonance imaging was deferred due to acute kidney injury (serum creatinine 2.1 mg/dL, baseline 0.9 mg/dL), which precluded gadolinium administration. Endocrinopathies, such as thyroid disease, were excluded based on normal thyroid function tests. No infective or neoplastic etiology was identified on imaging or laboratory tests. Collectively, the workup supported a diagnosis of PICM, particularly due to the high ventricular pacing burden and absence of alternative explanations. After excluding all these differentials and considering the temporal association with pacemaker implantation, PICM became the most plausible diagnosis.

She required intensive care support for hypotension and respiratory distress secondary to large pleural effusions. Intravenous furosemide and temporary inotropic agents were initiated, and a right-sided intercostal drain yielded 1.6 L of pleural fluid, with no re-accumulation before pacemaker upgrade. Given the high RVP burden and significant decline in LVEF, the device was upgraded to a cardiac resynchronisation therapy pacemaker (CRT-P). The procedure was performed without contrast due to the coexisting renal impairment. Post-upgrade device interrogation confirmed >98% biventricular pacing with QRS narrowing from 180 ms to 140 ms.

**Figure 3:**
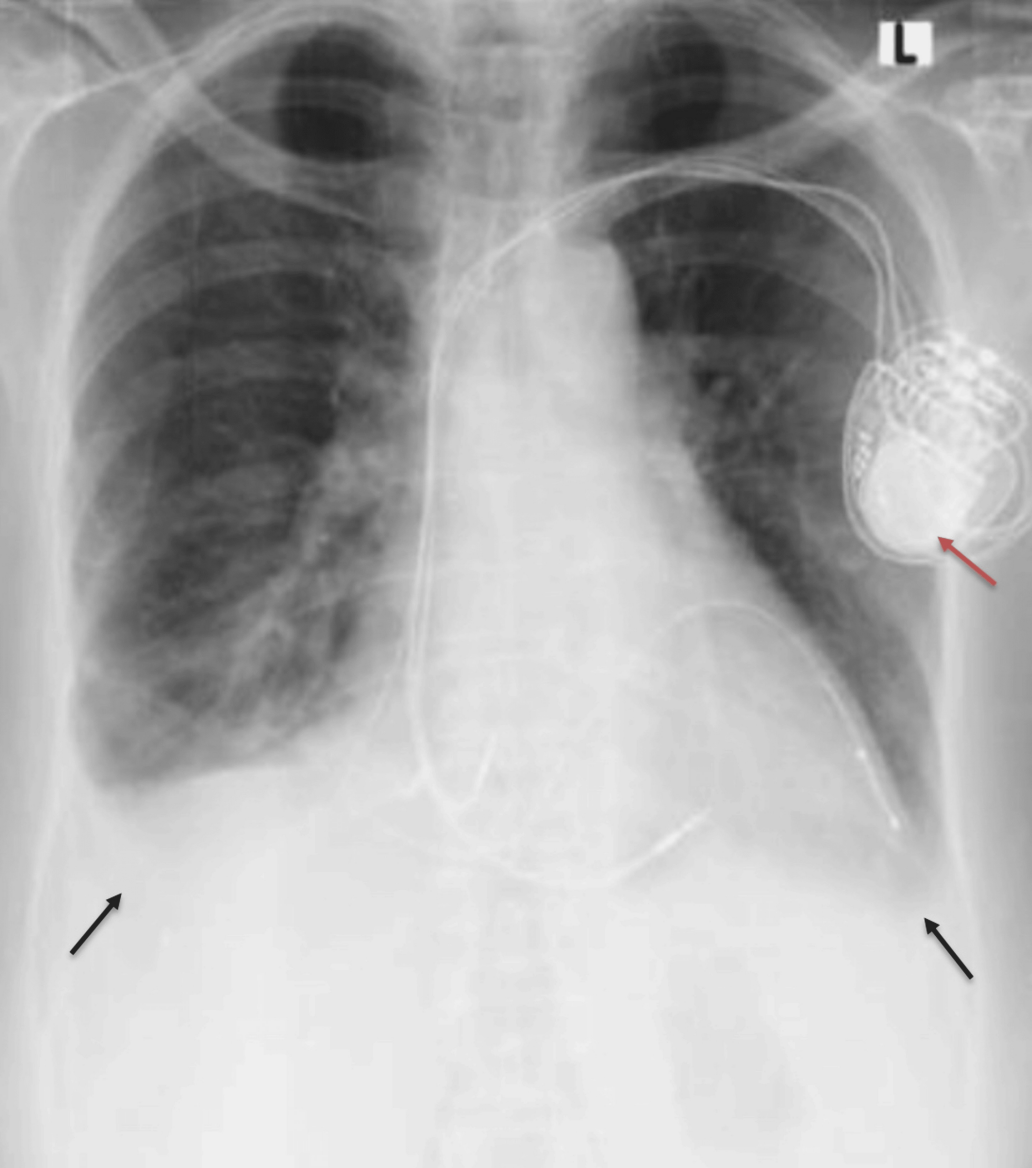
Repeat frontal chest radiograph post cardiac resynchronisation therapy pacemaker (CRT-P) insertion Demonstrates reduction in bilateral pleural effusions (black arrows) and newly inserted CRT-P (red arrow)

Following CRT-P implantation, the patient demonstrated rapid haemodynamic improvement. Guideline-directed medical therapy (GDMT) for heart failure with reduced ejection fraction was initiated, comprising bisoprolol 2.5 mg daily, enalapril 2.5 mg twice daily, empagliflozin 10 mg daily, and spironolactone 25 mg daily, with doses titrated as tolerated. Blood pressure stabilised, and she was weaned off inotropic support within 12 hours. Intercostal drainage was discontinued by postoperative day 3, with radiographic resolution of the pleural effusion by day 5 (Figure 3). Serum creatinine improved to 1.4 mg/dL with cautious diuresis and avoidance of nephrotoxins.

She was discharged in a stable condition on optimal GDMT. At four-week follow-up, she reported a return to baseline functional capacity (NYHA class I). Echocardiography showed an improved LVEF of 45%, and NT-proBNP had decreased to 4,966 pg/mL. These findings supported the diagnosis of pacing-induced cardiomyopathy and demonstrated the therapeutic efficacy of CRT-P upgrade.

## Discussion

PICM is an increasingly recognised complication of chronic RVP, particularly in patients who are highly pacemaker-dependent [1,2]. PICM is commonly defined as a reduction in LVEF of ≥10% to a value below 50% in the setting of high-burden RVP and the absence of other identifiable causes of cardiomyopathy [6]. The underlying mechanism is believed to stem from electrical dyssynchrony caused by non-physiological activation of the ventricles, which leads to adverse remodelling, interventricular and intraventricular dyssynchrony, and ultimately, impaired systolic function [6]. As seen in this case, PICM can develop insidiously and present with features of progressive heart failure several months after PPM implantation.

The diagnosis of PICM is primarily clinical and one of exclusion. It requires careful consideration of the patient’s timeline of symptom onset, exclusion of alternative causes of cardiomyopathy, and correlation with pacing burden and echocardiographic findings [7]. In this patient, the absence of ischemic, valvular, infectious, infiltrative, or metabolic causes strengthened the likelihood of PICM as the underlying diagnosis.

However, it is essential to acknowledge that the role of cardiac resynchronisation therapy (CRT) as a preventive strategy in all pacemaker candidates remains controversial. The BLOCK-HF trial demonstrated that biventricular pacing reduced the risk of heart failure hospitalisation or death in patients with AV block and reduced LVEF. Still, its findings were limited to a specific subset of patients with pre-existing systolic dysfunction [8]. In contrast, the BIOPACE trial did not show a significant benefit of routine biventricular pacing over conventional RV pacing in a broader population of patients with normal or mildly reduced LVEF. It was associated with a higher rate of complications [9]. These results caution against indiscriminate use of CRT in all pacing candidates.

Management of PICM is centred around reversing dyssynchrony. CRT has emerged as the mainstay of treatment in patients with PICM who exhibit significant left ventricular systolic dysfunction [10]. CRT restores more physiological electrical activation by simultaneous pacing of both ventricles or by targeting the conduction system, which improves cardiac output, mitigates remodelling, and often leads to symptomatic and functional recovery [11]. In this case, the CRT upgrade was associated with rapid hemodynamic improvement, resolution of pleural effusions, and significant recovery of left ventricular function within four weeks. These outcomes underscore the effectiveness of timely CRT intervention in reversing PICM-related deterioration. Despite advancements in pacing technology, PICM remains underdiagnosed, particularly in resource-limited settings where routine echocardiographic monitoring may not be consistently implemented. This highlights the importance of structured follow-up for patients with PPMs, particularly those with high ventricular pacing burdens. A structured follow-up plan should include baseline and periodic echocardiographic assessment (e.g., at six to 12 months post-implantation and thereafter), combined with device interrogation to identify patients at risk of PICM for early intervention [6,12].

In light of these limitations, conduction system pacing (CSP) has emerged as a promising physiological alternative to conventional RVP. CSP strategies, including His bundle pacing (HBP) and left bundle branch area pacing (LBBAP), aim to preserve native conduction pathways and prevent the onset of pacing-induced dyssynchrony [12]. Several observational studies and small-scale trials have demonstrated that CSP may result in better preservation of LVEF, lower rates of PICM, and improved clinical outcomes compared to traditional RVP. Furthermore, multiple randomised controlled trials are currently underway to evaluate the efficacy of CSP in reducing the incidence of PICM and heart failure admissions (e.g., HOPE-HF, LBBP-RESYNC). While long-term outcome data are still pending, CSP holds significant potential for altering future pacing paradigms [13,14].

This case also emphasises the importance of a multidisciplinary approach, involving cardiologists, electrophysiologists, and intensivists, to ensure early recognition and prompt intervention in patients with new-onset heart failure post-pacemaker implantation.

## Conclusions

PICM is a potentially reversible yet often underrecognized complication of chronic right ventricular pacing. This case highlights the clinical significance of vigilant post-implantation monitoring and the importance of early intervention when signs of cardiac dysfunction appear. Upgrading to CRT can result in significant symptomatic and functional improvement. A structured follow-up strategy, combined with awareness of pacing-related complications, is crucial for improving long-term outcomes in patients with permanent pacemakers.

However, while CRT remains the cornerstone of treatment in established cases, its role as a preventive strategy is limited, with trials such as BLOCK-HF and BIOPACE showing mixed outcomes. Emerging alternatives such as CSP, including His bundle and left bundle branch area pacing, offer promising physiological benefits and may reduce the risk of PICM, though long-term evidence is still evolving.
